# Fulminant myocarditis associated with human rhinovirus A66 infection: a case report

**DOI:** 10.3389/fped.2024.1480724

**Published:** 2024-10-28

**Authors:** Shuaibing Han, Jing Liu, Ziheng Feng, Yiyang Mao, Hengmiao Gao, Zhengde Xie, Suyun Qian, Lili Xu

**Affiliations:** ^1^ Key Laboratory of Major Diseases in Children, Ministry of Education, National Clinical Research Centre for Respiratory Diseases, National Key Discipline of Paediatrics (Capital Medical University), Beijing Paediatric Research Institute, Beijing Children’s Hospital, Capital Medical University, National Centre for Children’s Health, Beijing, China; ^2^ Research Unit of Critical Infection in Children, Chinese Academy of Medical Sciences, Beijing, China; ^3^ Department of Paediatric Critical Care Medicine, Beijing Children's Hospital, Capital Medical University, National Centre for Children's Health, Beijing, China

**Keywords:** human rhinovirus, fulminant myocarditis, virus myocarditis, child, immunotherapy, case report

## Abstract

**Background:**

Human rhinoviruses (HRVs) are among the most common pathogens of upper respiratory infections, and they are responsible for the common cold. An increasing number of studies have shown that HRV is associated with more severe illness. However, HRV-associated fulminant myocarditis has rarely been reported.

**Patient presentation:**

A previously healthy 8-year-old boy developed fever, fatigue, and vomiting for 3 days, with a subsequent exacerbation accompanied by confusion lasting for 9 h. The day before admission, the patient presented with oliguria, confusion, and hypotension, and he was suspected of having myocarditis. The patient was transferred to our hospital for further diagnosis and treatment. On admission, rough and moist rales were detected, and the heart sounds were muffled, accompanied by an irregular heart rhythm and a gallop. An electrocardiogram (EKG) revealed a wide QRS complex, ST-segment depression, premature ventricular contractions, and complete right bundle branch block. Laboratory tests revealed that brain natriuretic peptide (BNP), N-terminal pro BNP (NT-pro BNP), and cardiac biomarkers, such as troponin I, creatinine kinase (CK), and creatinine kinase-MB (CK-MB) were elevated. Additionally, echocardiography revealed an ejection fraction of approximately 28%. The child developed severe cardiac dysfunction and tissue hypoperfusion, and the cardiogenic shock could not be corrected despite active drug therapy. He had indications for ECMO implantation. A rarely reported rhinovirus, namely, A66, was detected in his bronchoalveolar lavage fluid and oropharyngeal swabs via metagenomic next-generation sequencing and a PCR assay. Bacterial culture of all the samples yielded negative results.

**Conclusions:**

This case presents a patient with severe human rhinovirus A66 infection, which is likely responsible for fulminant myocarditis. This report facilitates prompt diagnosis and treatment of fulminant myocarditis. Clinicians should consider rhinovirus as a possible pathogen of fulminant myocarditis, especially when patients present with symptoms or signs of heart involvement.

## Background

Human rhinovirus (HRV) is classified in the *Enterovirus* genus within the *Picornaviridae* family (1, 2). HRV is a common viral aetiology of upper respiratory infections and is mostly associated with cold and flu-like illnesses (3–5). In most cases, HRV infection is mild and self-limiting (1, 4–6). Until recently, accumulating evidence has indicated that these viruses are associated with more severe diseases, such as recurring wheezing, asthma, and exacerbations of chronic obstructive pulmonary disease (COPD), as well as severe bronchiolitis in infants and children and fatal pneumonia in elderly immunocompromised patients (1, 7, 8). In addition, severe extrapulmonary complications are increasingly recognized (5, 8). We previously reported a severe case of HRV-A45 with central nervous system involvement and viral sepsis in a 10-year-old girl (9).

Myocarditis is an inflammatory disease of the myocardium (10–12), and the course of this disease is self-limiting in most patients (12). Myocarditis can be caused by various infectious and noninfectious agents. Among the infectious agents, viruses are the most common cause. Thes clinical presentation of myocarditis varies from subclinical to clinical to fulminant myocarditis (FM). Patients with FM present with rapid haemodynamic decompensation, cardiogenic shock, arrhythmias, and multiorgan system failure. Myocarditis should be suspected in children who have clinical signs of heart failure, increased cardiac biomarkers, and/or abnormal left ventricular function, especially when there is a recent history of viral illness. Therefore, rapid diagnosis and timely treatment are important for the prognosis of a patient (12).

Until now, the factors that determine whether patients present with fulminant or nonfulminant myocarditis have been unclear. Previous studies have shown that myocarditis is caused by various agents, including infectious pathogens (viruses, parasites, bacteria, fungi, chlamydia, rickettsia, and protozoans), toxins, various drugs, and systemic illnesses (12, 13). Among all agents, myocarditis in children is most often caused by virus infection. The commonly identified pathogens of myocarditis are listed in Table 1 (12, 14). Among these viruses, coxsackievirus B3 (CVB3) and adenovirus (AdV) are the most common, but they also include parvovirus B19 and human herpes virus 6 (10–12, 15, 16). However, few studies have reported HRV-associated myocarditis. A previous study has revealed that HRV is a rare cause of acute-onset dilated cardiomyopathy due to myocarditis in a newborn (17). Another study has indicated that the HRV detection rate is the highest in children with myocarditis (16).

**Table 1:** Aetiologic agents of viral myocarditis.

	RNA viruses	DNA viruses
Virus type	Coxsackie virus A and B Echovirus Severe acute respiratory syndrome coronavirus 2 (SARS-CoV-2) Poliovirus Respiratory syncytial virus Mumps virus Rubella virus Dengue virus Yellow fever virus Influenza A and B virus	Adenovirus Cytomegalovirus Herpes simplex virus Varicella virus Parvovirus B19 Epstein‒Barr virus Human immunodeficiency virus

Herein, we present a severe case of fulminant myocarditis associated with HRV infection. HRV infection was confirmed by metagenomic next-generation sequencing (mNGS) and PCR.

## Case presentation

Here, we report the case of an 8-year-old boy with no previous cardiac history or remarkable personal or family history. The patient had recurrent fever without apparent inducement 3 days before admission, with the highest body temperature of 39.4°C, accompanied by fatigue, vomiting, and abdominal pain. On the day prior to admission, he developed oliguria, confusion, and hypotension.

**Figure 1:**
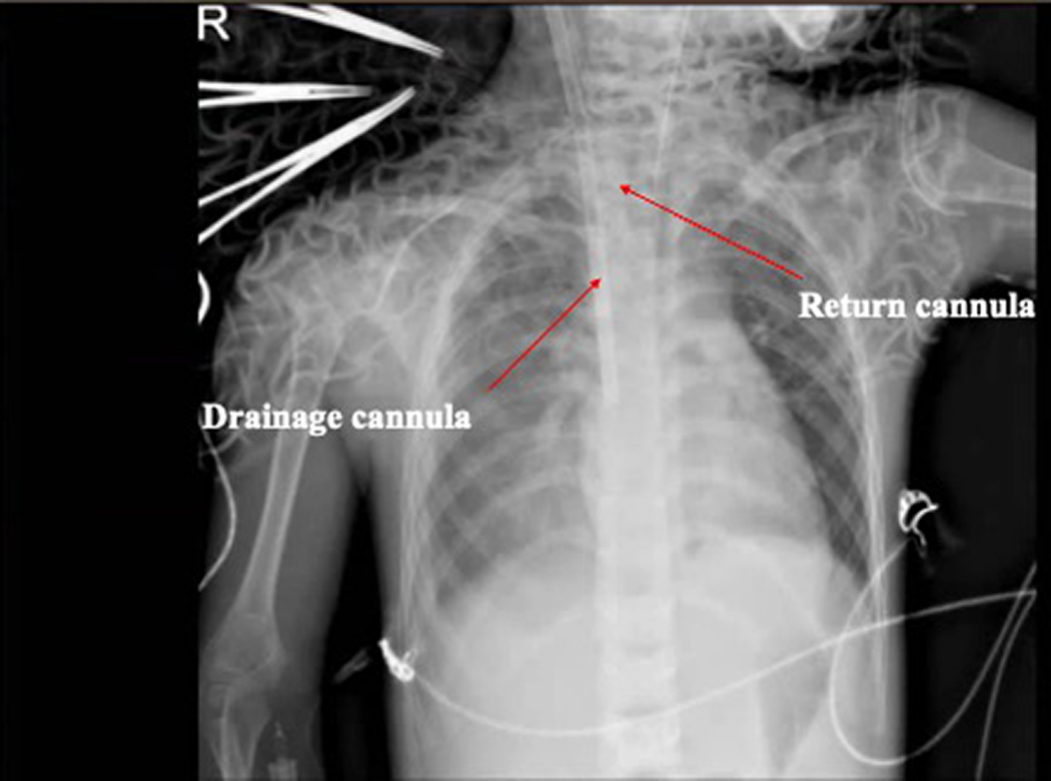
ECG from the day of admission revealed widening of the QRS complex, ST-segment depression, arrhythmia (premature ventricular complexes), and complete right bundle branch block.

**Figure 2:**
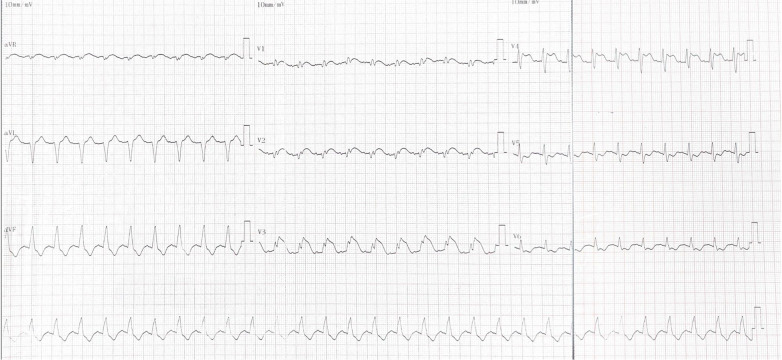
Radiographic examination of the chest. The images revealed that the texture of both lungs increased, thickened, and blurred, and patchy shadows were observed in the right lower lung. The ECMO cannulas are shown in the figure with arrows.

Upon admission, his exam revealed bilateral rales, muffled heart sounds, and an audible gallop. A chest x-ray revealed a high-density shadow over the lower field of both lungs, a large heart shadow, and bilateral pleural effusion (Figure 1). Serum laboratory investigations revealed significantly elevated C-reactive protein (CRP), brain natriuretic peptide (BNP), and NT-pro BNP levels. Cardiac biomarkers, including creatinine kinase (CK), CK-MB, and troponin I, were elevated (Table 2). Electrocardiography (ECG) revealed widening of the QRS complex, ST-segment depression, premature ventricular complexes, and complete right bundle branch block (Figure 2). Echocardiography revealed a significant reduction in cardiac function and ejection fraction (EF, 28%), with mildly decreased right ventricular systolic function, mild mitral regurgitation, and mild/moderate tricuspid regurgitation. Additionally, the internal diameters of the atria and ventricles were not significantly enlarged. The interventricular wall and the left ventricular posterior wall were not markedly thickened (Table 3).

**Table 2:** Changes in the main laboratory indices of the patient after admission.

Date	CRP (mg/L)	WBC (10^9^/L)	HB (g/L)	PLT (10^9^/L)	PCT (ng/ml)	LDH (U/L)	*α*-HBDH (U/L)	ALT (U/L)	AST (U/L)	CREA (μmol/L)	CK (U/L)	CK-MB (ng/ml)	BNP (pg/ml)	HSTNI (ng/ml)	LAC (mmol/L)
2022.11.02	26	10.54	123	252	–	–	–	745.8	1333.6	49.6	1,700	128.3	660.7	>50	6.1
2022.11.03	21	12.7	111	198	17	3,567	2,022	1,748	2883.5	84.8	–	32.2	1697.1	31.75	2.2
2022.11.04	16	37	97	150	7.6	1,924	1,298	1506.3	1375.2	87.2	907	7.4	1669.8	25.603	0.8
2022.11.05	–	–	–	–	–	–	–	–	–	–	–	2.3	916.6	18.2	0.7
2022.11.06	<10	18.1	104	109	1.6	1,316	893	1099.6	488.3	55	–	1.6	2279.1	9.2	0.6
2022.11.07	15	15.6	101	138	0.5	790	687	–	–	–	–	–	–	–	0.6

WBC, leukocytes; HB, haemoglobin; PLT, platelet; PCT, procalcitonin; LDH, lactate dehydrogenase; α-HBDH, α-hydroxybutyrate dehydrogenase; ALT, alanine aminotransferase; AST, aspartate aminotransferase; CREA, creatinine; TnI, troponin I; CK-MB, creatine kinase; BNP, B-type natriuretic peptide; LAC, lactis; “–”, no data.

**Table 3:** Echocardiographic data of the patient after admission.

Date	LA	LV		RA		RV			
	Anteroposterior diameter (mm)	End-diastolic anteroposterior diameter (mm)	EF (%)	Left-right diameter (mm)	Superior-inferior diameter (mm)	Anteroposterior diameter (mm)	Left-right diameter (mm)	TAPSE (mm)	FAC (%)
2022.11.02	19.5	35.9	20	30.8	29.8	13.4	28.1	13.9	35
2023.01.09	18.0	35.7	65	Normal	9.5	−	−	−	−

LA, left atrium; LV, left ventricle; RA, right atrium; RV, right ventricle; EF, ejection fraction; TAPSE, tricuspid annular plane systolic excursion; FAC*,* functional aerobic capacity; –, no data.

The patient presented to the emergency department in cardiogenic shock. To support his cardiovascular system and optimize organ perfusion, he was started on an infusion of epinephrine and dobutamine, and he was endotracheally intubated. Cardiogenic shock management requires myocardial support with agents that improve contractility and do not increase systemic vascular resistance because the increase in systemic vascular resistance adds more stress to the failing left ventricle. The common combination of agents is low-dose epinephrine (beta 1 adrenergic agonist) +/− milrinone (phosphodiesterase inhibitor). Although dobutamine is not commonly used in the paediatric setting, the rationale in this case was related to its effect on contractility (beta 1 agonist) without increasing SVR. However, the haemodynamic status of the patient continued to deteriorate, requiring emergent cannulation and venoarterial extracorporeal membrane oxygenation (V-A ECMO) for cardiovascular support. Epinephrine and milrinone were infused because an echocardiogram revealed severely impaired cardiac function, with an ejection fraction of approximately 20% (Table 3). An electrocardiogram revealed supraventricular tachycardia and a broad abnormal waveform with no obvious sinus *P* wave, and his heart rate was 170 beats per minute, suggesting that amiodarone should be administered for antiarrhythmic intervention. As the heart rate gradually decreased, it transitioned back to sinus rhythm, and amiodarone was no longer administered.

On the second and third days after admission, the child underwent continued treatment with pulse therapy with methylprednisolone. On the fourth day after admission, cardiac echocardiography revealed an EF of 40%. The ECMO flow rate and blood flow gradually decreased. By the sixth day after admission, sinus rhythm was evident on the electrocardiogram. The second ECMO weaning trial was successful. The patient remained on VA ECMO support for a total of 6 days. He was extubated and transitioned to nasal continuous positive airway pressure (NCPAP) on hospital Day 9. His cardiac function continued to improve, and he was discharged home on hospital Day 16. His outpatient follow-up at 5 months post discharge revealed normal cardiac function (EF 65%).

## Viral analysis

### mNGS for pathogen detection

Bronchial alveolar lavage fluid (BALF) and serum samples were collected from the patient on the first day of admission to our hospital for metagenomic analyses via the NGS platform. Nucleic acids were extracted from all samples via TRIzol LS reagent (Thermo Fisher Scientific, Carlsbad, CA, USA) and a Direct-zol RNA miniprep kit (Zymo Research, Irvine, CA, USA) following the manufacturer's instructions. DNA and RNA concentrations were measured with a Qubit fluorometer (Thermo Fisher Scientific). DNA libraries were constructed through transposase-mediated methods, followed by PCR amplification (Life Technologies, Carlsbad, CA). The quality of the DNA/RNA libraries was assessed via a Qsep1 Bio-Fragment analyser (BiOptic, La Canada Flintridge, CA) to measure the sizes of the fragments before sequencing. The quantified libraries were pooled and sequenced on a NextSeq 550Dx sequencing platform (Illumina). A single-end (75-bp read length) sequencing strategy was used. Reads that passed these filters were mapped against human references via Bowtie v2.2.4. Reads that aligned with either of the references were removed. The remaining reads were subjected to a BLASTn v2.2.30 search, and mapping was performed via CLC Genomics Workbench v21.0.3. All tools were run with default parameters unless otherwise specified. Human rhinovirus type A (HRV A) was identified from bronchoalveolar lavage fluid (3,540 reads). The random distributions of the reads of the HRV A sequences covered 99% of the total HRV A genome. Torque teno virus 3 (TTV3) was also detected in the bronchoalveolar lavage fluid (4 reads). Because approximately 40%–50% of healthy individuals can carry TTV3 and the number of TTV3 reads was low, this virus was not considered to be related to this case. Furthermore, mNGS analysis of bronchoalveolar lavage fluid revealed opportunistic pathogenic bacteria, including *Staphylococcus aureus*, *Streptococcus constellatus*, *Streptococcus anginosus*, and *Pseudomonas aeruginosa*. The blood culture results were negative for bacterial growth, and the patient's clinical presentation was not indicative of a bacterial infection.

### Viral molecular analysis

**Figure 3:**
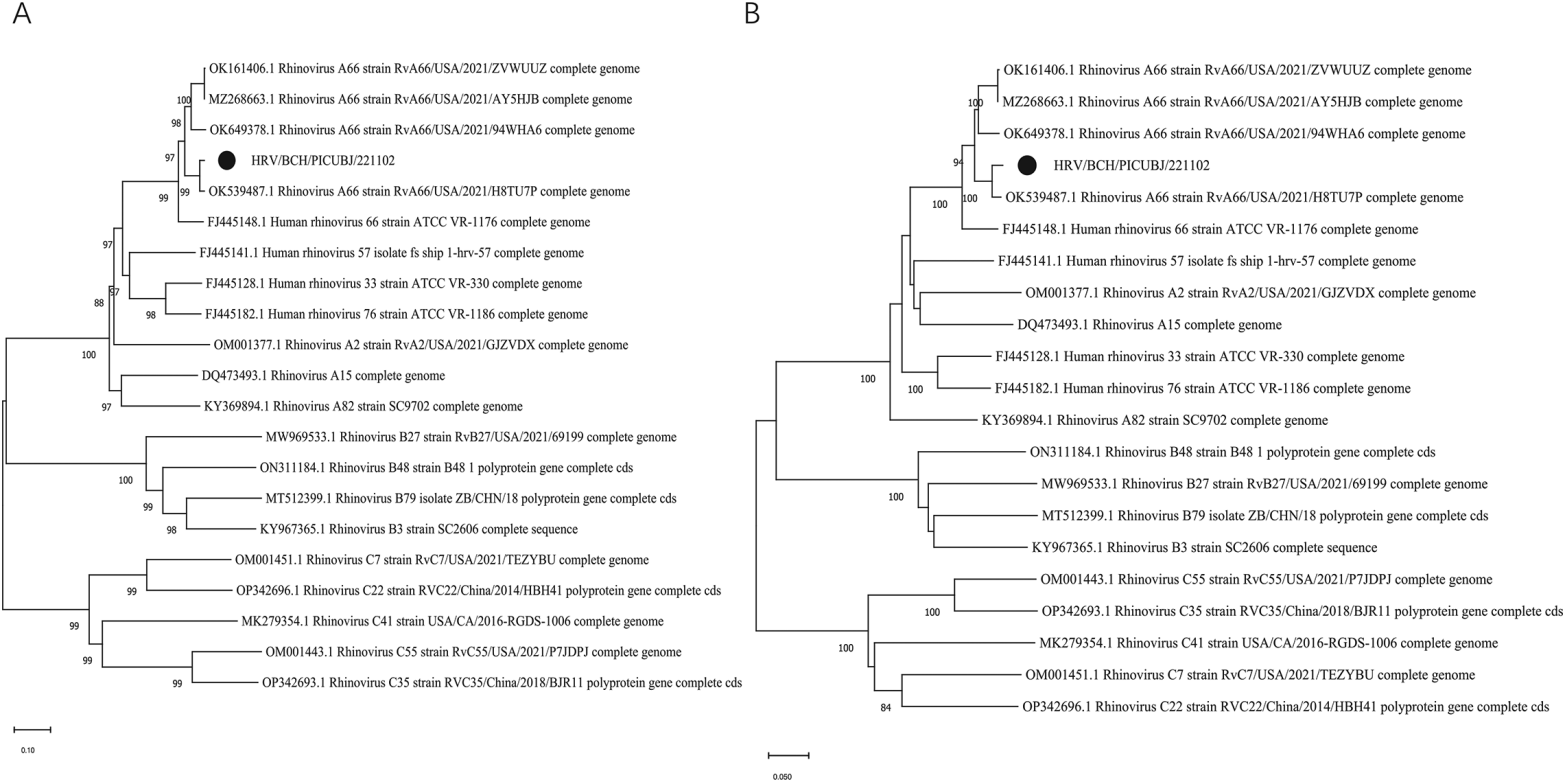
Phylogenetic analysis of HRV-A66 detected in this patient and other representative sequences of HRV. MEGA 11.0 software was used to construct phylogenetic trees via the neighbour-joining method. The phylogenetic robustness trees were assessed via the bootstrap method with 1,000 replicates via the same software. The strain in the present study is marked with a solid black circle. (**A)** Phylogenetic tree based on the completed genome sequences. **(B)** Phylogenetic tree based on the VP1 gene sequences.

Conventional reverse transcriptase PCR (RT–PCR) targeting the VP4/VP2 region of HRV-A was used to verify the mNGS results as previously described (18). The complete length of the HRV A genome was obtained via next-generation sequencing (NGS). The HRV strain was subtyped as HRV-A and named HRV/BCH/PICU/BJ221102, and the complete genome sequence was deposited in GenBank (accession number: OR801306). Multiple-sequence alignments were constructed with MAFT (https://www.ebi.ac.uk/Tools/msa/MAFFT/) software (version 7.407) via the accuracy-oriented method (L-INS-i). Phylogenetic trees based on the complete genomes and the viral gene VP1 were generated via the neighbour‒joining method, and bootstrap values of MEGA software version 11.0 with 1,000 replicates were calculated to evaluate confidence estimates (Figure 3). Phylogenetic analysis revealed that the HRV-A strain in the present study was subgrouped into A66. Phylogenetic analysis and nucleotide homology comparisons revealed that this strain was closely related to four available reference sequences from GenBank (RvA66/USA/2021/94WHA6, RvA66/USA/2021/ZVWUUZ, RvA66/USA/2021/AY5HJB, and RvA66/USA/2021/H8TU7P) and the prototype strain (HRV 66 strain ATCC VR-1176). Moreover, compared with the ATCC VR-1176 HRV-66 prototype strain, 6 amino missense mutations were identified (N656S in VP2, Y906C in P2-A, T909I in P2-A, R1458K in P3-A, I1507V in P3-A, and S1706T in P3-C).

## Discussion

We report the case of an 8-year-old male with prodromal viral illness who presented with cardiogenic shock and was diagnosed with fulminant myocarditis associated with HRV-66 infection. HRV-A66 and several opportunistic pathogenic bacteria were identified in his bronchoalveolar lavage fluid via mNGS. However, the clinical presentation of this patient did not support bacterial infection, and the results of blood cultures were negative for bacterial growth.

HRVs are often associated with upper respiratory tract infection, sinusitis, and otitis media, and they are also known as “common cold virus” (5). Importantly, with the rapid advancements in molecular detection methods for HRV groups and strains, more is known about the spectrum of illness associated with HRVs (1). In addition to the entire respiratory tract, rhinovirus antigens can also be detected in blood, faeces, and cerebrospinal fluid (5, 9). Research has indicated that rhinovirus viremia is associated with a greater viral load and more severe disease (19). Previous studies have reported that myocarditis and dilated cardiomyopathy (DCM) are associated with rhinovirus, whereas the association between rhinovirus A and FM has rarely been reported (16, 17, 20, 21).

Myocarditis in children is commonly caused by viral infection, which is often preceded by respiratory or gastrointestinal symptoms (16). With the development of more recent molecular biological techniques, an increasing number of viruses have been identified as the cause of myocarditis. Rhinoviruses are among those mentioned as causal agents of few childhood cases in the literature (16, 17, 20, 21). Some studies have indicated that direct damage to cardiomyocytes caused by viral infection is particularly important in FM. There are three phases in viral myocarditis. The first phase is the initial viral illness without signs or symptoms of cardiac dysfunction. In this phase, the destruction of cardiomyocytes is caused by virus-mediated lysis and the innate immune response. During the second phase, namely, the inflammatory phase, autoreactive T cells are induced and destroy cardiomyocytes. Moreover, the cross-reactive antibodies, owing to the viral antigen and myocardial proteins containing the same or similar antigenic epitopes, also destroy cardiomyocytes. The third phase is the dilated cardiomyopathy (DCM) phase, in which the destroyed myocytes are replaced by diffuse fibrosis (22).

FM is clinically defined as cardiac inflammation, with rapid onset of heart failure and cardiogenic shock, and early diagnosis is extremely important for patients. Therefore, a high degree of clinical suspicion is needed when patients present with symptoms and signs. The children mostly have nonspecific prodromal symptoms, such as fever, chest tightness, abdominal pain, and vomiting. Some children also present with easy fatiguability, loss of appetite, shortness of breath, cough, wheezing, tachycardia, hypotension, and syncope. When patients experience acute haemodynamic changes, they experience serious arrhythmias and multivisceral involvement, and severe cases are accompanied by cardiovascular failure and cardiogenic shock (23). Therefore, when patients have symptoms of both respiratory infections and heart involvement, clinicians should consider using an extensive respiratory panel.

The diagnosis of myocarditis is intricate and relies on the clinical presentation, as well as findings from various diagnostic tests, including assessments of cardiac biomarkers, electrocardiography (ECG), transthoracic echocardiography, cardiac magnetic resonance imaging (cMRI), and endomyocardial biopsy (EMB) (12, 24). Fulminant myocarditis is considered the most severe manifestation of acute myocarditis, typically characterized by the abrupt onset of cardiac symptoms subsequent to nonspecific influenza-like symptoms, and it swiftly progresses to severe haemodynamic dysfunction with cardiogenic shock, severe heart failure, and potentially fatal arrhythmias. In the present case, laboratory data revealed elevated markers of inflammation and cardiac biomarkers (Table 2). Among these markers, troponin I has been reported to have a sensitivity of 34% and a specificity of 89% (12). Moreover, elevated lactate dehydrogenase (LD/LDH), α-hydroxybutyrate dehydrogenase (α-HBDH), or aspartate aminotransferase (AST) may also be used for the diagnosis of myocarditis (23). ECG findings in fulminant myocarditis patients may include sinus tachycardia, ST segment changes, T-wave changes, and left bundle branch block or heart block (12, 25). On echocardiography, fulminant myocarditis is distinguished by a nondilated left ventricle exhibiting severe systolic dysfunction and augmented wall thickness, which is indicative of the existence of myocardial oedema (12). In addition, the tricuspid annular plane systolic excursion (TAPSE) markedly decreases at the initial stage of the illness, which is related to functional decline in the right heart caused by sustained overload (26).

Myocarditis can be caused by infectious or noninfectious aetiologies, which are characterized by inflammation. Because immunoglobulins (IVIGs) have antiviral, anti-inflammatory, immune-modulating, and antioxidative stress effects, intravenous IVIG may be a potent immunomodulatory therapy for myocarditis (10, 22, 27, 28). Although IVIG is commonly used in children, it was not used in the present case. Thus far, the role of IVIG remains largely investigational in children with viral myocarditis (10, 29). Previous studies have indicated no significant treatment benefit in terms of mortality or improved LV systolic function from IVIG treatment for paediatric patients with acute myocarditis (10, 27). Another recent multicentre study has failed to reveal a survival benefit associated with IVIG therapy for paediatric myocarditis (27). In addition, studies have noted that high-dose steroids constitute another immune-modulating therapy that may have a therapeutic effect on paediatric myocarditis (27). However, recent reviews and meta-analyses have reported that high-dose steroid therapy does not significantly impact clinical outcomes (27). In addition, steroids are not routinely used in myocarditis, but they have been used more frequently since the SARS-CoV-19 pandemic in patients with MIS-C (30). Moreover, to date, no approved pathogen-directed or antiviral therapies for patients with viral myocarditis are available (10).

The cause of myocarditis after rhinoviral infection has not been elucidated. However, both immune-mediated and direct cytotoxic mechanisms of the myocardium may play important roles in this pathogenesis, similar to heart diseases caused by other viruses. As a member of the immunoglobulin superfamily, coxsackie-adenoviral receptor (CAR) has adhesion molecule functions and serves as a versatile internalizing receptor for the complete range of coxsackie B virus family and diverse enteroviral viruses (25, 31). The levels of immunologic activation markers, such as intercellular adhesion molecule-1 (ICAM-1), soluble FAS ligands, and T cell activation markers, are increased in patients with myocarditis (25). Previous studies have indicated that most HRV-A serotypes enter the cell via ICAM-1 or bind low-density lipoprotein receptor (LDL-R) (6, 32), which is potentially the critical step for viral infection. The presence of cross-reactive antibodies indicates that the viral antigen shares antigenic epitopes that are similar to those found in myocardial proteins (25). Cardiomyocyte damage can be directly induced by viruses. In parallel, virus-mediated humoural and cell-mediated immunity play major roles in myocardial remodelling and in progressive heart failure (25).

As previously stated, the occurrence of extra-respiratory symptoms and severe disease in relation to HRV is infrequent (1, 5, 8). The present study reported the association of HRV A66 with FM. Genomic variation may be a determinant of viral adaptation and pathogenesis. In the present case, 6 missense mutations were identified in the strain, which were located in VP2, P2-A, P3-A, and P3-C. Previous research has demonstrated that amino acid mutations occurring in the EF loop region of VP2 are significant neutralization sites within picornaviruses and that aa mutations in these regions may change the ability to neutralize the virus (33). Proteases 2A and 3C cleave viral polyproteins, and protein 3A plays a crucial role in anchoring replication complexes to the membranous structure. Moreover, in addition to its role in proteolytic cleavage of viral polyproteins, 3C plays a crucial role in antiviral immune responses (5). However, further studies are required to determine whether the mutations in the present strain are associated with fulminant myocarditis.

The present study had several limitations. The present study only detected HRV in the BALF and oropharyngeal swabs but failed to provide further evidence, such as the associations of serum IgM antibodies against HRV, serum IgG, or total antibodies with HRV. The nucleic acid analyses suggested the presence of HRV in the upper respiratory tract. If the serum IgM antibody against HRV is positive and if the titre of serum IgG or total antibody against HRV doubles or increases to even higher levels, this may further support the present conclusion. However, because the samples were stored for a long period, there was a failure to the serum IgM levels. Recently, cardiac magnetic resonance imaging (cMRI) has evolved as the gold standard for noninvasive diagnostic imaging in myocarditis patients because of its ability to detect inflammation, oedema, necrosis, and fibrosis within myocardial tissue (10, 34). This patient did not undergo cMRI given his fulminant presentation and the need for emergent ECMO cannulation upon arrival. Moreover, as the gold standard for the diagnosis of myocarditis, endomyocardial biopsy (EMB) is conducive to the discovery of pathogens and the investigation of pathogenesis. However, EMB is limited by its invasive nature and sampling error; for younger children, the risk of arrhythmia and other complications is significantly increased when EMB is conducted during the acute phase (23, 35).

In summary, a case of fulminant myocarditis associated with HRV-A66 was presented. Thus far, no studies have reported HRV A-associated fulminant myocarditis. The results of the present study emphasized the significant role of HRV infection in fulminant myocarditis. The present report highlighted the importance of identifying early signs of cardiac dysfunction and recognition of heart failure, especially viral myocarditis, which should be considered when a patient simultaneously has a history of any virus infection. Therefore, the present study facilitates prompt diagnosis and treatment, thereby improving the prognosis of fulminant myocarditis.

## Data Availability

The raw sequence data of the case reported in this study has been deposited in the Genome Sequence Archive (Genomics, Proteomics & Bioinformatics 2021) in National Genomics Data Center (Nucleic Acids Res 2022), China National Center for Bioinformation/Beijing Institute of Genomics, that are publicly accessible at https://ngdc.cncb.ac.cn under the BioProject of PRJCA031180.
